# Case series: Interstitial lung disease with IPF pattern presenting with spontaneous pneumothorax—clinical course of two patients

**DOI:** 10.3389/fmed.2025.1664024

**Published:** 2025-10-15

**Authors:** Amit Toshniwal, Babaji Ghewade, Alushika Jain, Saket Satyasham Toshniwal

**Affiliations:** Datta Meghe Institute of Medical Sciences, Wardha, India

**Keywords:** interstitial lung disease, idiopathic pulmonary fibrosis, spontaneous pneumothorax, pneumomediastinum, subcutaneous emphysema

## Abstract

**Background:**

Interstitial lung disease (ILD) encompasses a broad spectrum of fibrosing pulmonary conditions. Pneumothorax is a recognized complication of fibrotic ILD, but simultaneous occurrence with subcutaneous emphysema and pneumomediastinum is exceedingly rare.

**Case presentation:**

This case series describes two patients with idiopathic pulmonary fibrosis (IPF)-pattern ILD who experienced complications from spontaneous pneumothorax. In the first case, the patient arrived at the emergency department with worsening shortness of breath. Imaging revealed the presence of spontaneous pneumothorax, pneumomediastinum, and subcutaneous emphysema. The patient’s condition deteriorated rapidly and ultimately succumbed to respiratory failure. The second case involved a female patient who initially presented with chronic respiratory symptoms and was later diagnosed with probable usual interstitial pneumonia (UIP)-pattern ILD. During the follow-up, she exhibited worsening symptoms and was diagnosed with a spontaneous pneumothorax, which was treated successfully with conservative management.

**Conclusion:**

To the best of our knowledge, this is the first reported case series that document the triad of subcutaneous emphysema, pneumomediastinum, and pneumothorax in a patient with fibrotic ILD. The findings underscore the importance of timely recognition and multidisciplinary management for such high-risk patients.

## Introduction

Interstitial lung disease (ILD) refers to a diverse range of chronic pulmonary conditions characterized by varying degrees of inflammation and fibrosis. Idiopathic pulmonary fibrosis (IPF) is one of the most severe forms of ILD, which often results in a progressive decline in lung function and can lead to respiratory failure. The median survival rate after diagnosis of IPF is typically 3–5 years (1, 2). Spontaneous pneumothorax is a known but uncommon complication of ILD and is associated with increased morbidity and mortality. Studies have shown that pneumothorax in ILD, particularly in cases associated with connective tissue diseases, is an independent predictor of poor prognosis (3). Additional complications such as pneumomediastinum and subcutaneous emphysema, while individually documented, are rarely seen concurrently.

Air-leak syndromes in ILD typically result from alveolar rupture due to tractional stress on fibrotic tissue or as a consequence of immunosuppressive therapy (4, 5). While isolated cases of pneumothorax or pneumomediastinum have been described (6, 7), the co-occurrence of all three pneumothorax, subcutaneous emphysema, and pneumomediastinum has not been previously reported in a single patient with fibrotic ILD.

This case series highlights two distinct clinical courses in patients with fibrotic ILD. The first case demonstrates a rapidly progressive and fatal air-leak syndrome, while the second case presents a manageable pneumothorax during follow-up. The discussion integrates findings from recent literature, including the significance of radiological phenotypes such as pleuroparenchymal fibroelastosis (PPFE) in determining prognosis and clinical outcomes (8).

## Case 1

A 46-year-old man, a known case of interstitial lung disease (ILD) on long-term home oxygen therapy and antifibrotic treatment with nintedanib (150 mg orally, twice daily), presented to the emergency department with worsening shortness of breath and dry cough that persisted for 2 months duration. He also reported new-onset drowsiness, chest discomfort at rest, and an increased respiratory rate for 1 day prior to presentation.

His significant past medical history included cholelithiasis, choledocholithiasis, and common bile duct stricture, for which he had previously undergone angioembolization. During the clinical examination, he was drowsy but responsive, and he was disoriented to time, with a pulse rate of 118 beats per minute, a respiratory rate of 40 cycles per minute, blood pressure of 108/66 mmHg, and oxygen saturation of 91% on room air. His body mass index (BMI) was 23.4 kg/m^2^. Auscultation of the lungs revealed bilateral rhonchi and Velcro-like crackles.

Arterial blood gas (ABG) analysis at presentation revealed hypoxemia with a PaO₂ of 58 mmHg and PaCO₂ of 54 mmHg, indicating type II respiratory failure. Despite having an SpO₂ reading of 91% on room air, the elevated carbon dioxide level explained the patient’s drowsiness and disorientation, which are symptoms consistent with CO₂ narcosis. In patients with advanced ILD and chronic hypoxemia, adaptive tolerance to low oxygen saturation is common; however, acute hypercapnia due to decompensation can result in altered mental status. These findings warranted an urgent ICU admission for respiratory support.

**Figure 1:**
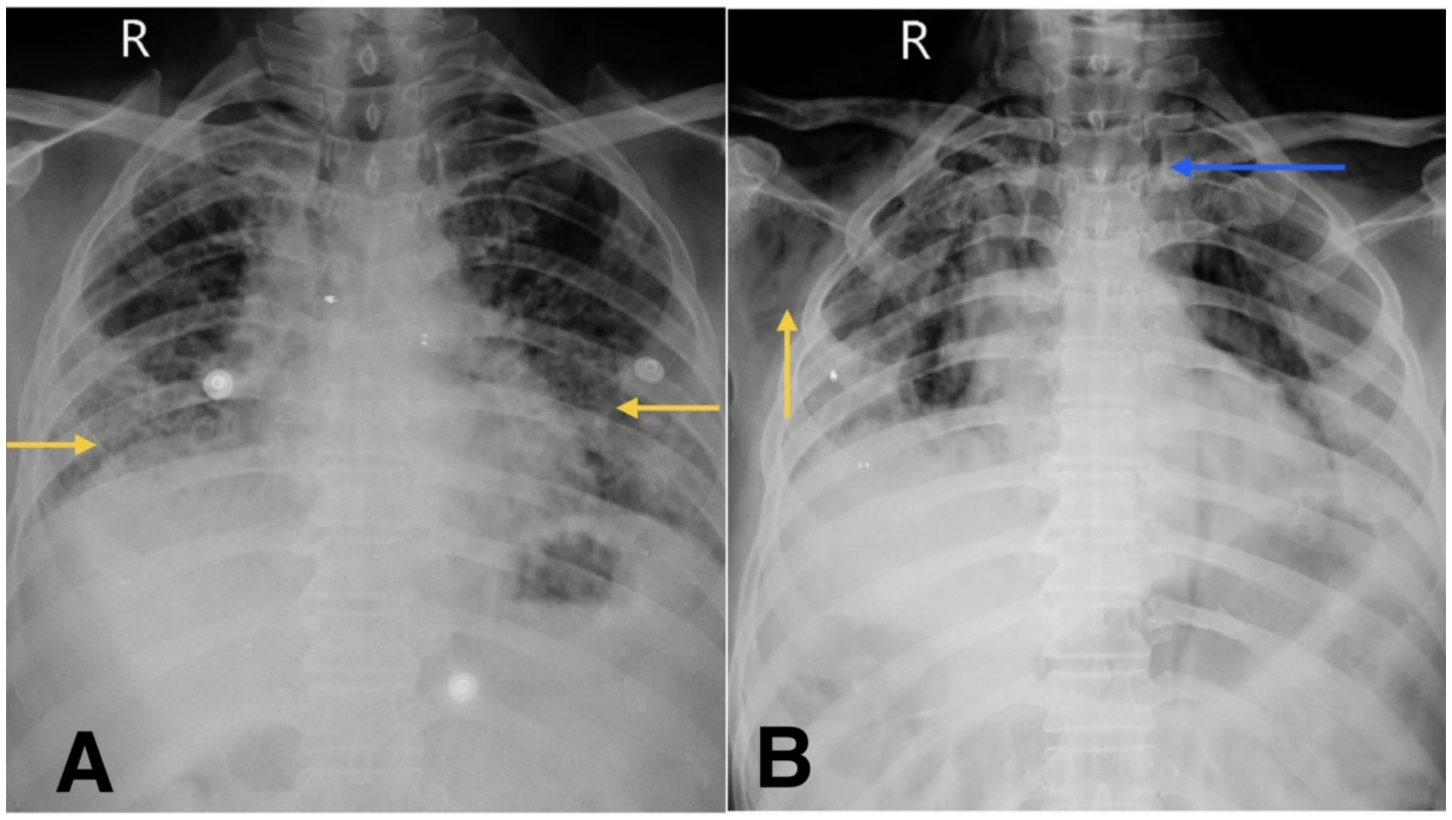
**(A)** Chest X-ray shows reticular opacity in bilateral lower zones of lung (yellow arrow). **(B)** Chest X-ray on follow-up visit reveals subcutaneous emphysema (yellow arrow) and suspicious pneumomediastinum (blue arrow).

**Figure 2:**
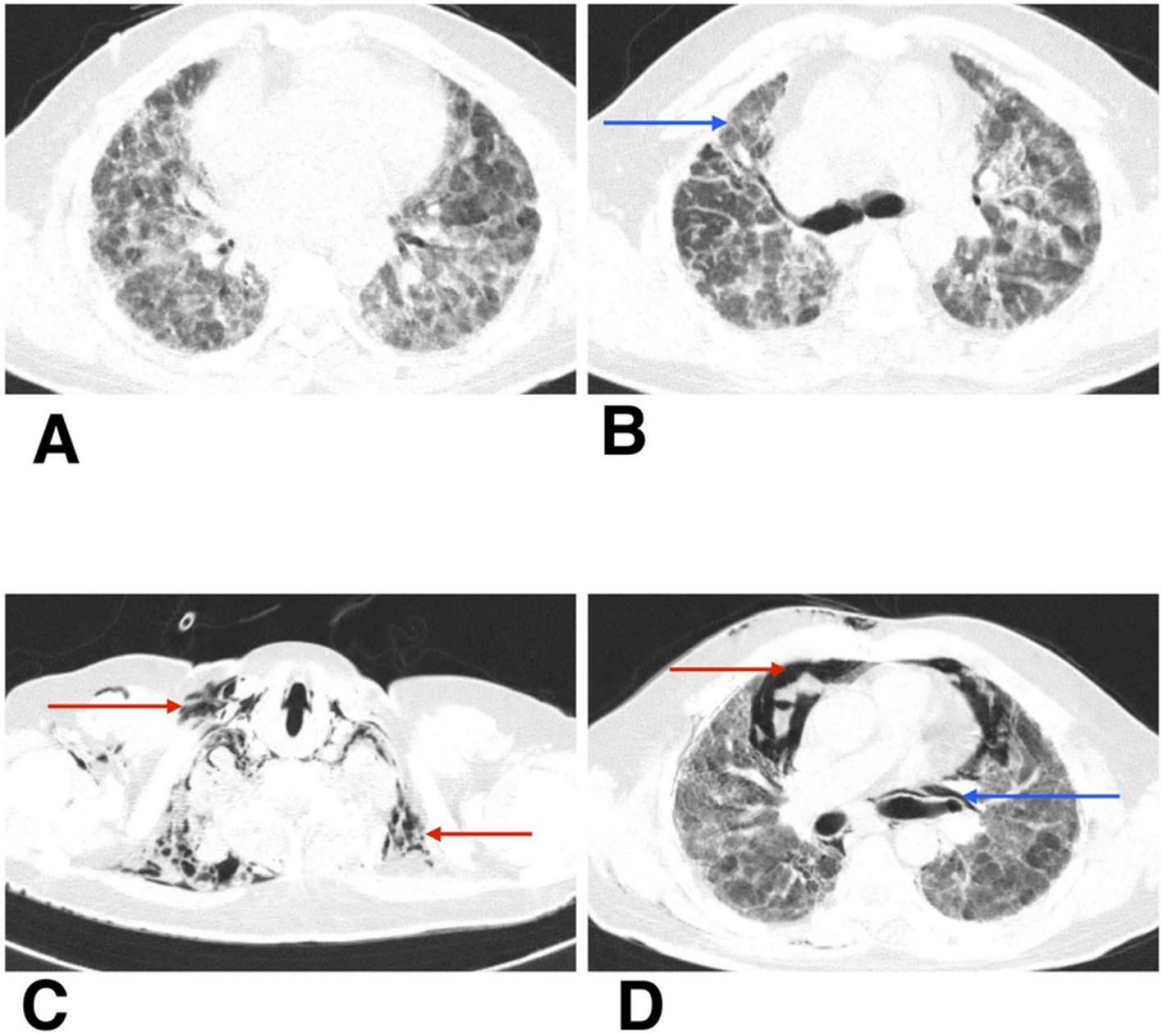
**(A,B)** HRCT thorax of axial section shows patchy ground-glass opacities, reticulations, and areas of architectural distortion (blue arrow). **(C,D)** HRCT thorax of axial section on follow-up shows subcutaneous emphysema (red arrow), spontaneous pneumothorax (red arrow), and pneumomediastinum (blue arrow).

He was admitted to the respiratory medicine department and transferred to the intensive care unit (ICU) for close monitoring. Chest radiography revealed reticular opacities in the bilateral lower lung zones (Figure 1A). High-resolution computed tomography (HRCT) of the thorax revealed findings consistent with fibrotic non-specific interstitial pneumonia (NSIP), including patchy ground-glass opacities, reticulations, and areas of architectural distortion (Figures 2A,B).

Microbiological analysis of the bronchoalveolar lavage (BAL) fluid showed the growth of *Candida krusei*. Smear for acid-fast bacilli (AFB) and GeneXpert *Mycobacterium tuberculosis*/rifampicin resistance (MTB/RIF) assays were negative.

He was managed with bilevel positive airway pressure (BiPAP) support using IPAP of 14 cm H₂O and EPAP of 6 cm H₂O, with FiO₂ titrated to maintain SpO₂ above 92%. Broad-spectrum intravenous antibiotics were initiated, including meropenem (1 g every 8 h for 10 days), azithromycin (1 g once daily for 5 days), and doxycycline (100 mg IV twice daily for 10 days). Systemic corticosteroid therapy was started with intravenous methylprednisolone (40 mg twice daily for 5 days), later tapered using oral prednisolone. Adjunctive supportive care included Duolin and Budecort nebulizations, vitamin C, mucolytics, sliding scale insulin, and nutritional supplements.

At the time of ICU admission, there was no clinical or radiological evidence of air-leak syndromes such as pneumothorax, subcutaneous emphysema, or pneumomediastinum. These complications were first identified following acute clinical deterioration.

During his first admission, the patient was managed with oxygen therapy, BiPAP support, and the treatment regimen detailed above. He improved clinically and was discharged with advice for long-term oxygen therapy at home and continuation of prescribed medications. However, after 1 month, he presented again to the emergency department with clinical deterioration. On this second presentation, he developed crepitus over the chest wall and neck, prompting urgent radiological reassessment. A chest X-ray revealed subcutaneous emphysema and features suspicious for pneumomediastinum (Figure 1B). Repeat HRCT thorax confirmed the presence of right-sided spontaneous pneumothorax, pneumomediastinum, and subcutaneous emphysema (Figures 2C,D).

Despite intensive care and escalation of respiratory support, the patient’s condition continued to worsen, culminating in death due to respiratory failure.

**Figure 3:**
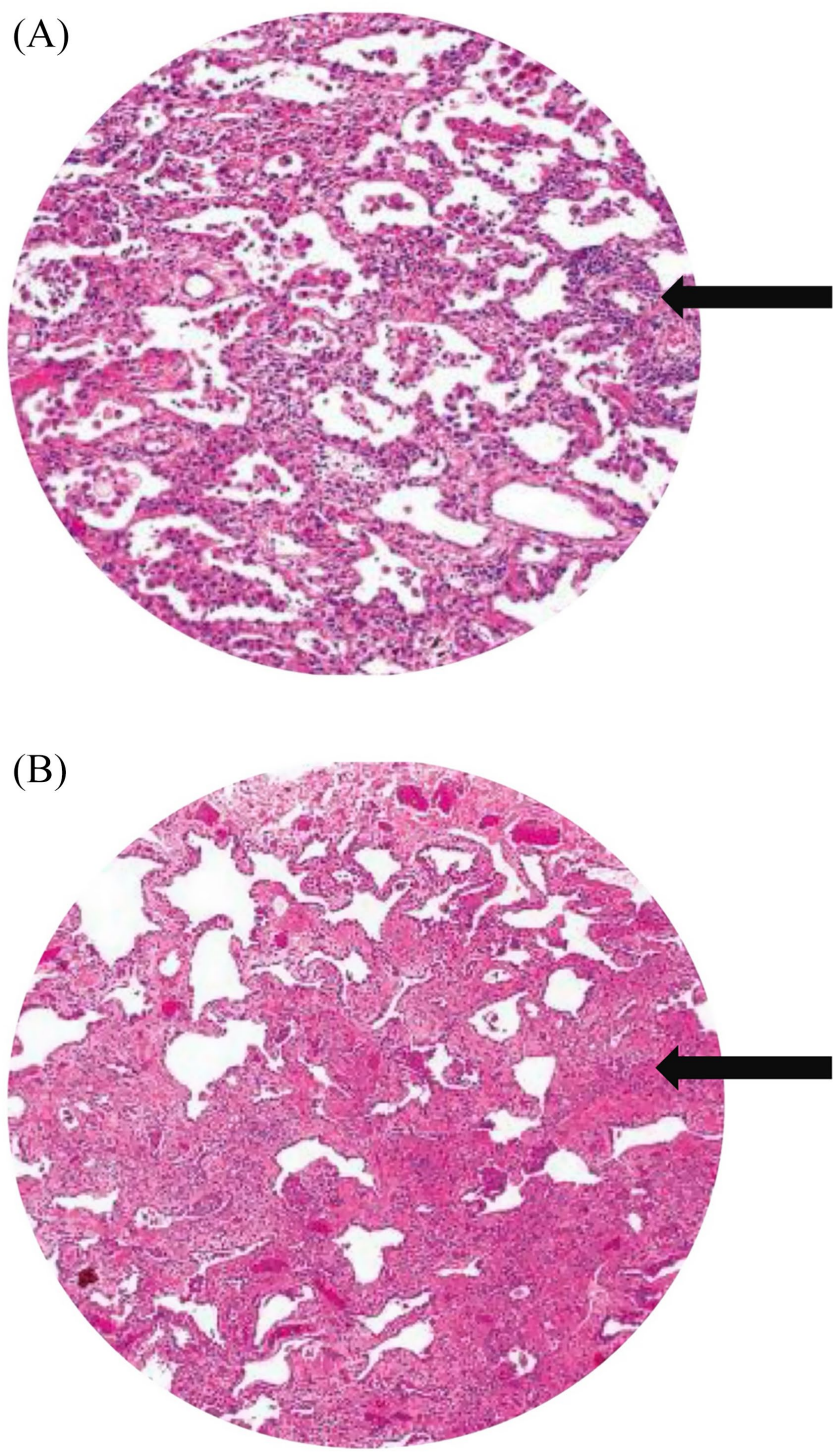
**(A)** Histopathological image shows cellular and fibrotic changes (black arrow). **(B)** Histology image shows dense collagenous fibrosis (black arrow), mild chronic inflammation.

Autopsy and clinicopathological correlation identified the cause of death as acute type I respiratory failure due to fibrotic non-specific interstitial pneumonia (fNSIP), characterized histologically by dense collagenous fibrosis with temporal uniformity, mild chronic inflammation, and relatively preserved alveolar architecture, and complicated by spontaneous pneumothorax, pneumomediastinum, and subcutaneous emphysema (Figures 3A,B).

## Case 2

The second patient was an female adult with a 2-month history of dry cough and progressive dyspnoea on exertion. Her symptoms were sudden in onset and were accompanied by significant constitutional symptoms, including the loss of appetite and weight. One month prior to admission, she had experienced an episode of haemoptysis. There was no history of prior pulmonary tuberculosis, hypertension, diabetes, or COVID-19 infection. On examination, she was hemodynamically stable and afebrile with a pulse rate of 96 beats per minute, blood pressure of 114/72 mmHg, respiratory rate of 24 breaths per minute, oxygen saturation of 94% on room air, and a BMI of 17.78 kg/m^2^. Respiratory examination revealed bilateral basal crepitations.

**Figure 4:**
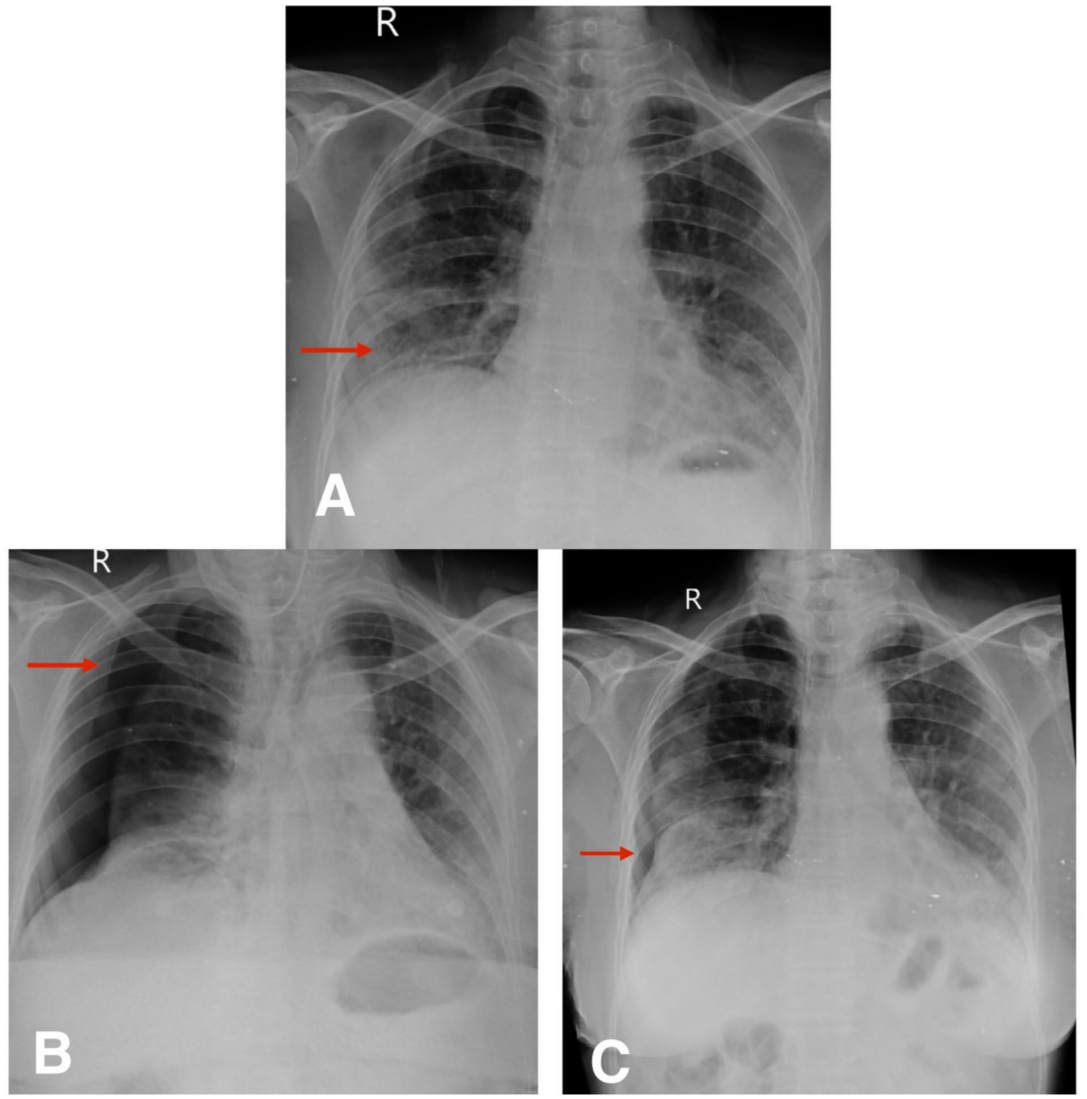
**(A)** Chest X-ray postero-anterior view shows reticular opacity in the right lower zone (red arrow). **(B,C)** Follow-up chest X-ray postero-anterior view shows spontaneous pneumothorax and slow resolution of pneumothorax (red arrow).

**Figure 5:**
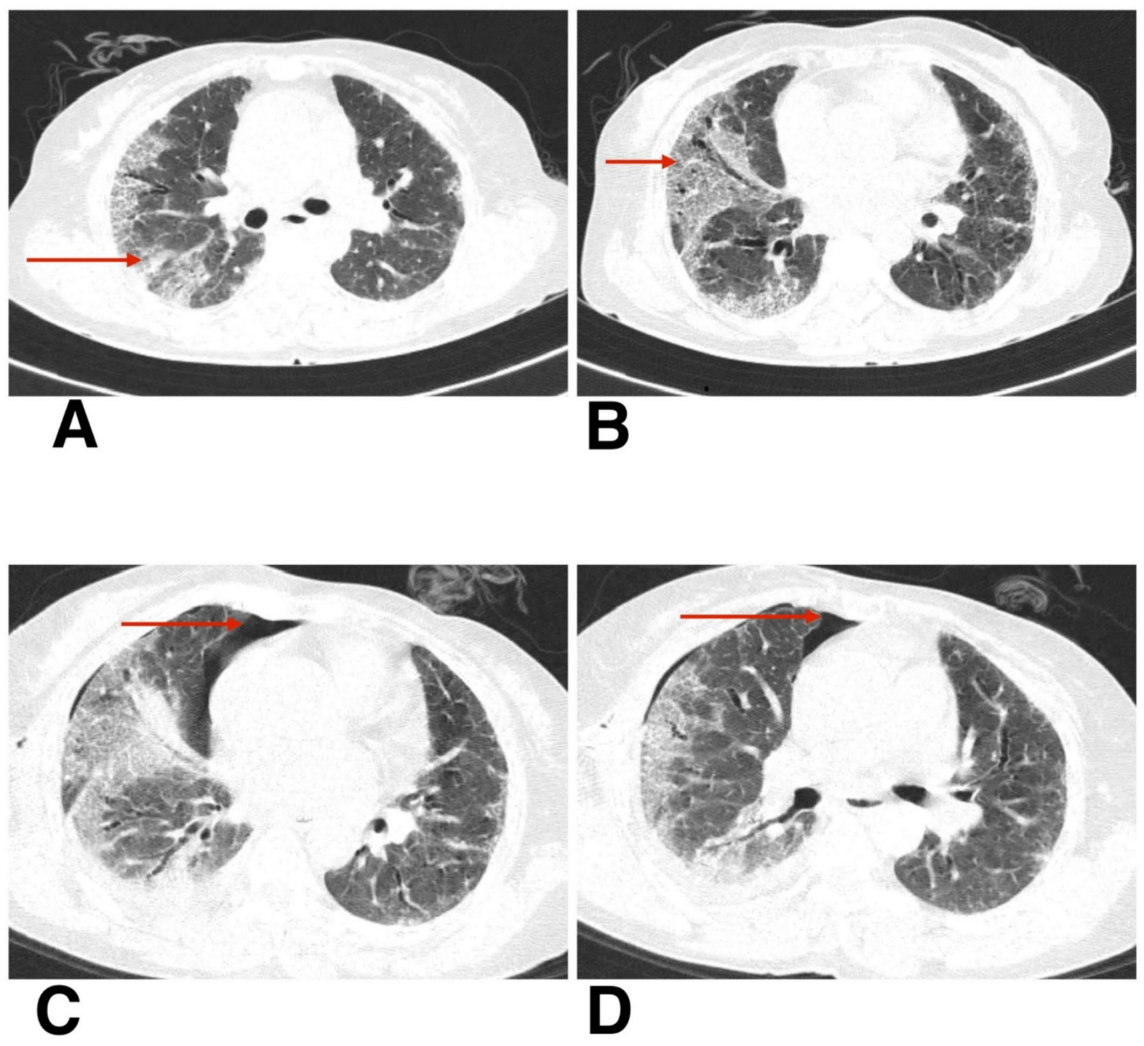
**(A,B)** HRCT thorax of axial section shows peripheral traction bronchiectasis, and a crazy paving pattern, consistent with a probable usual interstitial pneumonia (UIP) pattern. **(C,D)** HRCT thorax of axial section shows resolving pneumothorax after intercostal drainage and residual pneumothorax (red arrow).

Radiological evaluation with chest X-ray showed reticular opacity in the right lower zone (Figure 4A), and HRCT thorax demonstrates extensive subpleural and basal reticular opacities, peripheral traction bronchiectasis, and a crazy paving pattern, consistent with a probable usual interstitial pneumonia (UIP) pattern (Figures 5A,B).

A possible underlying systemic autoimmune rheumatic disease (SARD) was considered based on a mildly elevated antinuclear antibody (ANA) titer of 1:80 (speckled pattern), a high-sensitivity C-reactive protein (hsCRP) level of 12.6 mg/L, and rheumatoid factor (RA quantitative) of 28 IU/mL. Bronchoscopy with BAL showed a neutrophilic predominance (75% polymorphs) and the presence of gram-negative bacilli, though no pathogenic organisms were cultured. BAL cytology revealed reactive bronchial mucosal cells with macrophageal infiltrates but no malignancy. Sputum and BAL samples were negative for mycobacterial infection.

The patient was managed with oral nintedanib 100 mg twice daily, and intravenous hydrocortisone 100 mg three times daily, followed by a tapering course of oral prednisolone: 30 mg once daily for 5 days, then 20 mg for 5 days, and 10 mg for another 5 days. She also received azithromycin 500 mg once daily and amoxicillin-clavulanate (Augmentin) 625 mg three times daily. Bronchodilator support included Duolin nebulization three times daily and Budecort nebulization twice daily. Oral Mucinac 600 mg three times daily was administered as mucolytic therapy, along with ascorbic acid syrup (Ascoryl) 10 mL three times daily and lactulose syrup (Duphalac) 10 mL at bedtime for symptomatic relief.

To further contextualize our findings, we reviewed previously published case reports and series describing fibrotic ILD complicated by spontaneous pneumothorax, pneumomediastinum, and/or subcutaneous emphysema. Table 1 summarizes key characteristics from these cases, including ILD subtype, air-leak complications, management strategies, and outcomes. While several reports have documented individual occurrences of these complications, very few report two features simultaneously, and to our knowledge, none describe the triad seen in Case 1. This tabulated review enhances the clinical relevance of our series and highlights the spectrum of disease presentations and management outcomes across ILD subtypes.

**Table 1:** Summary of published cases of ILD with pneumothorax, pneumomediastinum, and/or subcutaneous emphysema.

Author (year)	ILD subtype	Complication(s)	Management	Outcome
Nishimoto et al., 2020 (3)	CTD-ILD	Pneumothorax	Pleurodesis, surgery	High mortality
Nadig and Thomson, 2011 (5)	Pulmonary fibrosis	Pneumomediastinum only	Conservative (O₂)	Recovered
Nishizawa et al., 2023 (6)	SSc-ILD + *M. abscessus*	Pneumothorax	Multidrug + Pleurodesis	Recovered
Subhadarshani et al., 2025 (7)	Anti-MDA5 dermatomyositis	Pneumomediastinum + Subcut Emphysema	Immunosuppression	Recovered
Present Case 1 (2025)	Fibrotic NSIP	Pneumothorax + Pneumomediastinum + SE	Supportive, O₂ (fatal)	Died
Present Case 2 (2025)	Probable UIP	Pneumothorax	Conservative	Recovered

### Follow-up

The second patient was hospitalized for 8 days during her initial presentation and treatment. Following discharge, she returned for a scheduled outpatient evaluation 2 weeks later. At this visit, she reported increased shortness of breath and chest discomfort. On presentation, her SpO₂ was 91% on room air. Supplemental oxygen was administered via nasal prongs at a flow rate of 4 L/min, titrated to maintain oxygen saturation above 94%. She remained hemodynamically stable throughout follow-up and did not require escalation to non-invasive or high-flow support. Chest X-ray confirmed the presence of a spontaneous pneumothorax (Figure 4B). Given her stable vitals and adequate oxygen saturation, she was managed conservatively with observation, supplemental oxygen, and supportive care. Follow-up chest X-rays over the next 2 weeks demonstrated gradual re-expansion of the lung (Figure 4C), and resolution of the pneumothorax was confirmed on high-resolution computed tomography (HRCT) (Figures 5C,D). The patient continues to be monitored at monthly intervals through outpatient visits, with adherence to antifibrotic and supportive therapy.

## Discussion

This case series describes two patients with fibrotic ILD. The first patient demonstrated radiological features consistent with fibrotic non-specific interstitial pneumonia (fNSIP), including lower lobe-predominant fibrosis, relative architectural preservation, and temporal homogeneity. The second patient exhibited a probable UIP pattern with subpleural reticulation and basal-predominant honeycombing. These complications, while individually documented in ILD, have not previously been reported in combination, making the first case an exceptional presentation in the literature to date.

The incidence of pneumothorax in fibrotic ILD varies across cohorts but is consistently associated with poor outcomes. In connective tissue disease-associated ILD, pneumothorax has been reported in up to 8–10% of patients, with median survival as low as 15 months following onset (3). In idiopathic pulmonary fibrosis, secondary spontaneous pneumothorax, while less common than in emphysema, has been documented as a recurrent and life-threatening complication. These data underscore the prognostic weight of air-leak syndromes in ILD populations.

Several mechanisms have been proposed to explain pneumothorax development in fibrotic lungs. Progressive tractional stress on fibrotic tissue can lead to alveolar rupture, especially in subpleural zones of honeycombing. Corticosteroid or immunosuppressive exposure may further weaken alveolar integrity, facilitating barotrauma even without mechanical ventilation. Chronic infection or colonization, as in our Case 1, may also contribute to alveolar fragility. Together, these mechanisms highlight the multifactorial nature of pneumothorax in ILD.

Management strategies range from conservative observation and oxygen therapy to invasive interventions such as intercostal drainage, pleurodesis, and surgical resection. Conservative management has been successful in select patients with limited pneumothorax and preserved function, as seen in Case 2. However, recurrence rates are high, and pleurodesis alone achieves durable success in only ~60–65% of cases. Surgical intervention, particularly VATS with bullectomy and pleurodesis, has demonstrated superior outcomes in eligible patients, with cure rates approaching 90–96% (4). Nonetheless, poor performance status or advanced fibrosis often precludes surgery, as in Case 1, necessitating reliance on supportive care. Preventive strategies, including antifibrotic therapy, early nutritional support, and judicious corticosteroid use, may also help mitigate risk in high-vulnerability patients.

Pneumothorax is a recognized complication in fibrotic ILD and has been consistently associated with poor outcomes. In a large study of 140 patients with connective tissue disease-associated ILD (CTD-ILD), Nishimoto et al. identified pneumothorax as an independent predictor of mortality (hazard ratio [HR] 22.0; 95% confidence interval [CI]: 10.3–46.9; *p* < 0.001), with a median survival of 15.2 months following onset (1). Both patients in this series exhibited radiologic hallmarks of fibrotic progression, including traction bronchiectasis and reticular opacities. Reticular changes of Grade ≥2 were associated with pneumothorax development (HR 2.41) and increased mortality (HR 2.48; *p* = 0.015) in the same cohort (3). In our series, Case 1 had a BMI of 23.4 kg/m^2^ (within normal range), while Case 2 had a low BMI of 17.8 kg/m^2^. Although low BMI has been reported as a risk factor for pneumothorax in ILD (HR 0.86; 95% CI: 0.73–1.02), the association in our series remains anecdotal, given the small sample size (as our observation is based on a single patient) (3). Further studies with larger cohorts are needed to validate this association.

Corticosteroid exposure is a known contributor to air-leak syndromes in ILD. The first patient received intravenous methylprednisolone prior to the onset of pneumothorax, pneumomediastinum, and emphysema. In the study by Nishimoto et al., methylprednisolone pulse therapy significantly increased the risk of pneumothorax (HR 3.40; *p* = 0.011) (3). Such immunosuppressive treatment may impair alveolar integrity and promote alveolar rupture in the setting of fibrotic distortion.

Surgical intervention remains the most definitive option for pneumothorax in ILD. In a study by Watanabe et al., 96% of patients who underwent surgery achieved a cure, whereas pleurodesis alone had a lower success rate (64.2%), particularly when performed more than twice (cure rate 62.5% vs. 81.0% for ≤2 attempts) (4). In our series, Case 1 had a PS of 4, which precluded surgical intervention, while Case 2 had a PS of 2 and was managed conservatively. This aligns with the findings of Watanabe et al., where PS ≥ 2 was associated with poor outcomes and limited treatment options (HR 4.05; *p* = 0.011), reinforcing the need for early surgical consultation (4).

Pneumomediastinum in ILD, although rare, has been reported in isolation. Nadig and Thomson described pneumomediastinum secondary to alveolar rupture in a patient with pulmonary fibrosis; however, the patient lacked coexisting subcutaneous emphysema or pneumothorax, and the condition resolved with conservative oxygen therapy (5). The first case in this series differs significantly, with all three air-leak complications occurring simultaneously and progressing fatally—an association not previously documented in published case reports to date.

Nishizawa et al. reported a case of systemic sclerosis-associated ILD complicated by *Mycobacterium abscessus* infection and secondary spontaneous pneumothorax. Treatment included multidrug therapy and pleurodesis, with eventual improvement (6). Although no bacterial pathogen was isolated in the first case here, bronchoalveolar lavage did yield *Candida krusei*, raising the possibility that colonization in a fibrotic, immunosuppressed lung may have contributed to alveolar fragility and barotrauma.

A recent report by Subhadarshani et al. described a case of anti-MDA5 dermatomyositis-associated ILD presenting with subcutaneous emphysema and pneumomediastinum. The patient responded to high-dose corticosteroids and azathioprine (7). However, no pneumothorax was reported in that case, distinguishing it from the fatal triad observed here. Furthermore, the simultaneous occurrence of pneumomediastinum, subcutaneous emphysema, and pneumothorax in the absence of trauma, mechanical ventilation, or overt infection appears to be unique.

Surgical intervention is increasingly recognized as the most definitive treatment for secondary pneumothorax in ILD, particularly when conservative methods fail. In a comprehensive retrospective study by Watanabe et al. (4) involving 103 patients with ILD and pneumothorax, 96% of those who underwent surgery experienced successful outcomes without recurrence, whereas the cure rate was only 64.2% in the pleurodesis group. Importantly, the number of pleurodesis procedures impacted the outcomes. Patients who underwent ≤2 pleurodesis procedures had an 81% success rate, compared to 62.5% in those receiving ≥3 sessions. The authors also found that a performance status (PS) score ≥2 was significantly associated with poor outcomes (HR 4.05; *p* = 0.011), highlighting the need to consider surgical options earlier in the treatment pathway (4).

Other studies have also supported the surgical approach in eligible patients. When patients have adequate pulmonary reserve and stable hemodynamic status, video-assisted thoracoscopic surgery (VATS) with procedures like bullectomy and pleurodesis has been associated with reduced recurrence and improved outcomes. However, in advanced ILD or patients with poor PS, as seen in Case 1, surgical intervention may be precluded, and outcomes remain poor despite aggressive supportive care.

These data underscore the importance of early multidisciplinary evaluation, including thoracic surgical consultation, in managing pneumothorax in ILD.

Management of pneumothorax in ILD differs from that in otherwise healthy patients, as limited pulmonary reserve, extensive fibrosis, and frequent comorbidities constrain therapeutic options. Standard strategies, such as oxygen supplementation, intercostal drainage, pleurodesis, and surgical intervention, must be tailored to the individual’s stability and performance status. In Case 2, conservative measures were sufficient due to her hemodynamic stability, smaller pneumothorax, and preserved PS (2). In contrast, Case 1 deteriorated rapidly with a triad of pneumothorax, pneumomediastinum, and subcutaneous emphysema on a background of fibrotic NSIP, severe hypercapnia, and poor PS (4), which excluded surgical or invasive options.

Prognostic divergence between the two cases likely reflects differences in baseline disease severity, treatment exposures, and systemic factors. Case 1 had advanced fibrosis, corticosteroid exposure, and Candida colonization, all of which may have predisposed to alveolar fragility and fatal progression despite preserved BMI. Case 2, although underweight (BMI 17.8 kg/m^2^) and diagnosed with probable UIP, had less extensive functional impairment, no hypercapnia, and responded favorably to conservative care with follow-up. Together, these cases emphasize that outcomes hinge on performance status, comorbidities, timing of intervention, and disease burden, and that early multidisciplinary evaluation is essential in guiding management.

In summary, this case series underscores that fibrotic ILD patients, particularly those with extensive reticulation, corticosteroid exposure, and low BMI, are at high risk of developing pneumothorax. In our series, Case 1 had a BMI of 23.4 kg/m^2^ (within normal range), whereas Case 2 had a BMI of 17.8 kg/m^2^, suggesting that while low BMI may contribute to risk, this association remains anecdotal and hypothesis-generating. The first case presented here is, to our knowledge, the first documented instance of subcutaneous emphysema, spontaneous pneumothorax, and pneumomediastinum occurring together in an ILD patient, highlighting a need for increased clinical vigilance and prompt multidisciplinary evaluation.

### Patient perspective

The family of the first patient expressed concern regarding the sudden and severe deterioration in his condition. Despite ongoing oxygen therapy and medication, they were unprepared for the rapid progression to respiratory failure. They appreciated the transparency and prompt updates provided by the ICU team.

The second patient reported feeling reassured by the timely follow-up care and radiological monitoring. She described her experience as initially distressing but felt optimistic after the pneumothorax was successfully managed without surgical intervention. She continues regular outpatient visits and remains compliant with her medical therapy.

## Data Availability

The raw data supporting the conclusions of this article will be made available by the authors, without undue reservation.
